# Spontaneous regression of a true splenic cyst: a case report and review of the literature

**DOI:** 10.4076/1757-1626-2-8730

**Published:** 2009-09-16

**Authors:** Christos N Stoidis, Basileios G Spyropoulos, Evangelos P Misiakos, Christos K Fountzilas, Panorea P Paraskeva, Constantine I Fotiadis

**Affiliations:** 1 3rd Department of Surgery, University of Athens Medical School, Attikon University Hospital, 1 Rimini Street, Chaidari, 12462, Greece; 2 Department of Internal Medicine, Athens Navy Hospital, 70 Deinokratous Street, Athens, 11521, Greece; 3 2nd Department of Propedeutic Surgery, University of Athens Medical School, Laikon General Hospital, 17 Agiou Thoma Street, Athens, 11527, Greece

## Abstract

Splenic cysts are rare clinical findings, detected due to derivative symptoms or as a random discovery in abdominal imaging. Although there still remains controversy as to their optimal treatment, bigger secondary cysts should be treated surgically. However, spontaneous regression may be observed in cysts with a diameter smaller than 4 cm. In these cases, expectant treatment is preferable. We report, herein, a single case of a splenic cyst in an adult woman, who reported minor symptoms despite the size of the lesion and who demonstrated a possible almost total regression of the cyst within a ten-year period, accompanying with review of the most recent literature.

## Introduction

Cystic lesions of the spleen have been recognized with increasing frequency since the advent of CT scanning and ultrasound imaging [1]. Splenic cysts are classified as true or false cysts, and they may be either nonparasitic or parasitic, and pseudocysts. Cyst-appearing tumors of the spleen include cystic lymphangiomas and cavernous hemangiomas. Primary true cysts of the spleen account for about 10% of all nonparasitic cysts of the spleen [2]. On the other hand, most nonparasitic cysts are pseudocysts and are secondary to trauma. The diagnosis of true splenic cysts is commonly made in the 2^nd^ and 3^rd^ decades of life [3]. True cysts are characterized by a squamous epithelial lining, and many are considered congenital. These epithelial cells are often positive for CA 19-9 and CEA by immunochemistry, and patients with epidermoid cysts of the spleen may have elevated serum levels of one or both of these tumor-associated antigens [4]. Despite the presence of these tumor markers, these cysts are benign and apparently do not have malignant potential greater than any other native tissue.

## Case presentation

A 42-year-old overweight woman from rural Greece visited the local physician in February 1997 reporting several episodes of mild upper abdominal pain during the previous three years, not related to food consumption. The pain was not observed elsewhere on the body nor was it correlated with other symptoms, such as fever, nausea or vomiting. In addition, the pain did not affect food intake or daily physical activity. Before medical consultation no systematic treatment had been applied for the pain, which was relieved after some minutes, only to relapse some days later.

**Figure 1:**
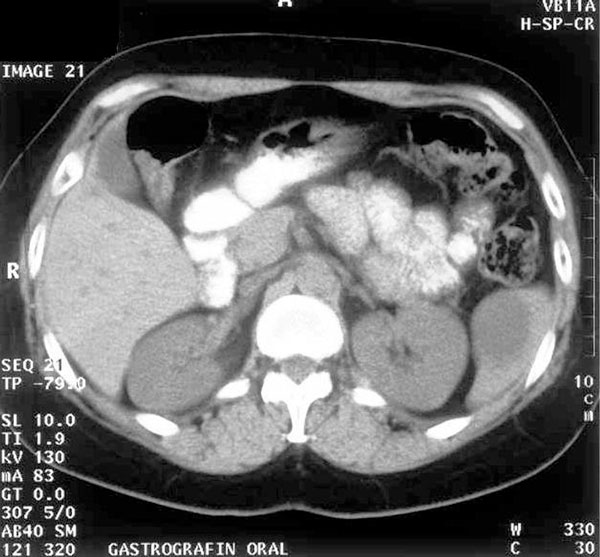
**CT scan (1997): A 6 cm splenic cyst is evident at the lower pole of the spleen**.

History revealed an episode of reported angina 4 months prior to consultation, with non -specific ST/T abnormalities in the ECG. Physical examination revealed no important findings. The blood tests revealed no obvious pathology, with a normal hematocrit and blood coagulation parameters. Biochemical testing for hepatic and renal was also within normal values. Imaging included a chest radiography, which revealed no abnormalities and an abdominal CT scan with oral gastrografin intake, which revealed a 3 × 6 cm cystic formation located at the lower pole of the spleen (Figure 1). Due to the lack of an infectious (no compatible clinical signs, no CRP or WBC count elevation, negative serological testing for Echinococcus) or traumatic background and the lack of evidence in favor of a cystic neoplasm (homologous content, normal perimeter, no increase in cancer-associated biochemical markers, such as Ca 19-9 and CEA), a latent congenital epithelial cyst was considered most probable. Four days later an MRI examination confirmed the previous result. The symptoms were not definitely associated to the cyst and the physician related them to the patient's increased stress, assuming she had irritable bowel syndrome.

**Figure 2:**
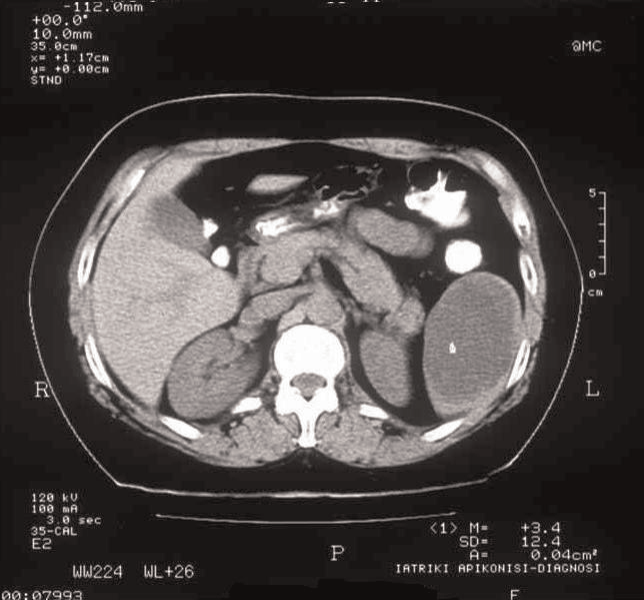
**CT scan (2005): A 3 × 5 cm splenic cyst is seen located at the lower splenic pole**.

**Figure 3:**
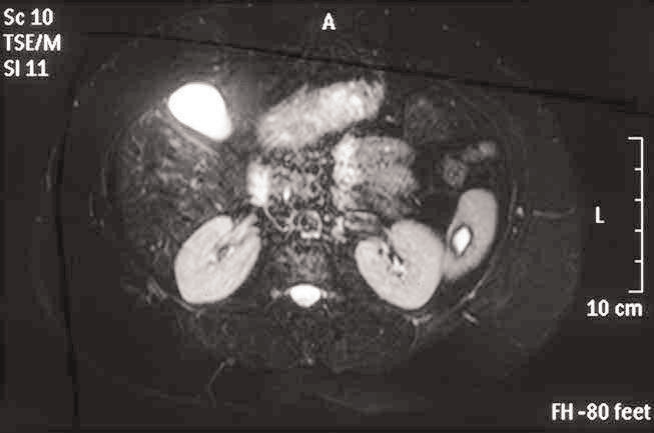
**MRI (2006): A 1**.8 × 1.4 cm cystic lesion has remained with a low T2 sign in the peripheral border.

Eight years later (2005), the patient visited her physician, reporting a periodic continuation of the abdominal pain, whereas neither the character nor the frequency of episodes differed. At this point, she was referred to the Attikon University Hospital for surgical treatment. An abdominal CT scan was performed as part of the preoperative evaluation, revealing a 3 × 5 cm splenic cyst which confirmed the existence of this random finding (Figure 2). The rest of the tests were within normal limits again. The patient decided to follow a conservative approach and re-evaluation at an annual basis. In November 2006, an ultrasound of the area revealed further regression of the cyst with a clear hyperechogenic perimeter, the size being estimated at less than 3 cm. For a more accurate description, an MRI scan was performed, which showed almost complete regression, with a remnant lesion of 1.8 × 1.4 cm and low T2 sign in the peripheral border (Figure 3), consistent with the pattern observed in the 2005 MRI (a comparative presentation of original and subsequent lesion is provided). Her last MRI examination in 2008 revealed the cyst dimensions equal to the last two radiographic images and a periodically regression of her nonspecific symptomatology, which finally proved that the presence of the cyst was responsible for her previous symptoms.

Surprisingly, in our case, the cyst, although particularly large, was only reported to cause mild symptoms though were not definitely linked to the finding itself, which may imply that former empirical treatment rules may not be applicable in all such cases after all.

## Discussion

Splenic cysts are generally uncommon clinical findings. They are classically categorized as primary and secondary. Primary (true) cysts have a complete epithelial wall, which may be related to infections (mostly parasites), neoplasms or congenital abnormalities. On the other hand, secondary cysts (pseudocysts) are mostly posttraumatic findings, but special infections and infracts are also likely causes. Although no complete registry of cases described so far is available, it has been estimated that about 800 cases have been diagnosed internationally. Of these, the large majority refers to parasitic cysts, although these are rare in developed countries. The remaining cases of non-parasitic cysts are about 300, described by numerous departments in small numbers [5]. The etiology of the splenic cysts is not always easy to determine. The general tendency is to exclude the usual and simpler diagnoses, i.e. infectious and traumatic lesions, before examining more rare possibilities, such as cystic neoplasms.

Often, true splenic cysts are asymptomatic and found incidentally. When symptomatic, patients may complain of vague upper abdominal fullness and discomfort, early satiety, pleuritic chest pain, shortness of breath, left back or shoulder pain, or urinary symptoms due to compression of the left kidney. A palpable abdominal mass may be present. The presence of clinical manifestation has been related to cyst position, type and size. As far as the latter criterion is concerned, scientific opinion varies, but 4 cm or bigger lesions are practically unanimously viewed as potentially symptomatic [6]. Rarely, these cysts may present with acute symptoms related to rapture, hemorrhage or infection. The diagnosis of splenic cysts is best established with CT imaging.

Symptomatic splenic cysts have been considered as high-risk lesions for future automatic rupture, which explains why most authors include them in the indications for surgical treatment (either splenectomy or more conservative operations) [7]-[9]. The treatment of splenic cysts is still a debatable issue in the relevant literature, referring to two independent questions:

i) Which cysts should be operated and which should be treated conservatively?

ii) Is open or laparoscopic approach the treatment of choice?

The answers provided so far have been based on recent clinical experience and concluded that rupture is more likely in lesions exceeding 4-5 cm in diameter, thus setting this size as the minimal indication for asymptomatic cyst operative treatment [10]. As far as the approach is concerned, this is primarily based on the experience of the center and available means, but generally, laparoscopy, where available and applied at a routine basis, offers comparable results, limiting surgical time, complications and post-operative hospitalization of the patient [11,12].

Operative intervention is indicated for symptomatic cysts and for large cysts. Either total or partial splenectomy may provide successful treatment. If the cyst is very large and almost completely covered by splenic parenchyma, or if it is located in the splenic hilum, complete splenectomy is recommended, because of the risk of intractable bleeding from the spleen.

The clear advantage of partial splenectomy is the preservation of splenic function [13]. Preservation of at least 25% of the spleen appears sufficient to protect against pneumococcal pneumonia. Most recent reports describe successful experience with partial splenectomy, cyst wall resection, or partial decapsulation, which may be accomplished with either an open or laparoscopic approach [14]. Partial splenectomy is recommended, if the cyst is located at the poles of the spleen, or if the cyst cavity is deep, due to the higher risk of recurrence [15]. Incision of the splenic capsule and hemostasis is performed with the ultrasonic or the monopolar scissors. A more conservative option could be a partial cystectomy (unroofing) of the cyst. However, it has yet to be determined how much of the cyst wall should be resected, and whether unroofing should be partial or radical. It is supported that as much of the cyst wall as possible should be resected to prevent reclosure of the cyst [16].

In our case, neither patient history nor all further evaluation, i.e. clinical, laboratory and imaging alike, succeeded in attributing the cyst to any of the more possible causes. Thus, the exact etiology remains indeterminate and the idea of a congenital lesion remaining unnoticed for 5 decades, seems to be very possible.

The physical history of splenic cysts may include all imaginable scenarios, i.e. stability, growth, rupture or involution. The latter was the case in our patient. However, relevant experience has stated that this outcome is more probable in congenital cysts during fetal and neonatal life or in adults with non-parasitic cysts (especially pseudocysts) smaller than 5 cm and within an observation period of up to 36 months [17]-[19]. However, our current case report indicates that this clinical rule is not always accurate in the prediction of clinical outcome, since a ten-year follow-up showed no signs of rupture, despite the original size of the lesion.

Our finding is therefore unique, since it refers to a possibly latent congenital epithelial cyst of relatively large original dimensions, which still reached almost total regression within a 10-year period. This observation may lead to the conclusion that experience with splenic cysts is still limited and, thus, currently available guidelines and treatment algorithms may be updated and reformed, as more recent findings, including our own, may gradually challenge the clinical concepts applied so far, suggesting that the patient's symptomatology and the cyst location and not the size alone should be the main criteria considered for surgical treatment of a true splenic cyst, since it is obvious that an enlarged cyst will cause analogous symptoms. The clinical outcome of a cyst independent of the size that causes mild symptoms is not easy to predict and observation could be the golden standard.

## Consent

Written informed consent was obtained from the patient for publication of this case report and accompanying images. A copy of the written consent is available for review by the Editor-in-Chief of this journal.

## Competing interests

The authors declare that they have no competing interests.

## Authors' contributions

CIF was the patient's surgeons and has been involved in drafting the manuscript and revising it critically for important intellectual content. PPP, CKF, CNS, BGS and EPM have made contributions to conception and design. CNS contributed to the analysis and interpretation of data. All authors read and approved the final manuscript. All authors contributed equally to the final draft of the manuscript. CIF has given the final approval of the version to be published.
